# Accuracy of Astigmatism Calculation with the Barrett, Panacea, and enVista Toric Calculators

**DOI:** 10.3390/life13102009

**Published:** 2023-10-03

**Authors:** Astrid Lucero Espinosa Soto, Jimena Ceja Martínez, Rosario Gulias-Cañizo, Oscar Guerrero-Berger

**Affiliations:** 1 Department of Anterior Segment Surgery, Fundación Hospital Nuestra Señora de la Luz, Mexico City 06030, Mexico; 2 Centro Oftalmológico Mira, Mexico City 03840, Mexico; 3 Centro de Investigación en Ciencias de la Salud, Universidad Anahuac Mexico, Naucalpan de Juárez 52786, Mexico

**Keywords:** toric intraocular lens, toric IOL, Barrett, EVO, emmetropia verifying optical, enVista, panacea, astigmatism, residual astigmatism, centroid

## Abstract

Purpose: To evaluate residual refractive astigmatism using the Panacea and enVista toric calculators, compared to the gold-standard Barrett toric calculator. Design: A retrospective and comparative study was conducted in one center. Methods: We reviewed the medical records of all patients with a diagnosis of senile cataracts and regular corneal astigmatism, without previous corneal or intraocular surgery, who underwent phacoemulsification with implantation of a toric intraocular lens, who had pre- and postoperative corneal topography, biometry, and refraction measurements. Results: The frequency of preoperative astigmatism according to the axis was 70 (84%) eyes showing with-the-rule (WTR) astigmatism, 9 (14%) eyes with against-the-rule (ATR) astigmatism, and 1 (2%) eye with oblique astigmatism. Regarding astigmatism prediction errors, there were statistically significant differences between the enVista and Panacea calculators (median of 0.39, 0.18, and 0.52 for Barrett, enVista, and Panacea, respectively). The residual astigmatism prediction error centroid was similar for the Barrett and enVista toric calculators, and both were lower compared to the Panacea calculator (x-component *p* < 0.001). Conclusions: The enVista toric calculator incorporating the Emmetropia Verifying Optical (EVO) toric calculator provides similar results to the gold-standard Barrett calculator.

## 1. Introduction

Cataract surgery has had a growing demand in recent years due to increased life expectancy and the need for patients to maintain productivity for more years. Phacoemulsification is the standard of care for cataract surgery in the developed world since technological advancements have greatly improved efficiency and patient safety, and today, it is considered a refractive surgery, generating expectations regarding visual results and spectacle independence. Refractive results have greatly improved, especially in the last decade, due to decreased surgical complications and the improved technology of intraocular lenses (IOLs), among other factors. However, one of the critical factors for success is the adequate performance of accurate formulas for IOL calculation to achieve the best visual results.

Myopia, hyperopia, and astigmatism, also known as refractive errors, are common visual disturbances caused by variations in the size or shape of the eyes. These cause visual disturbances since the images appear blurry or out of focus. Myopia occurs when the anteroposterior axis of the eye is longer or the cornea is more curved than normal, so image focusing occurs before the retina. Hyperopia is the opposite of myopia, as the eye is shorter than normal, so images of distant objects focus behind the retina. Unlike myopia or hyperopia, astigmatism is due to corneal deformation, which causes poorly defined, blurred, or distorted vision at all distances. Astigmatism may coexist with myopia or hyperopia. The prevalence of refractive errors in the Mexican population is 24.8% for myopia, 21% for hyperopia, and 13.5% for astigmatism [1,2]. In patients with astigmatism who are candidates for cataract surgery, 86.8% have mild astigmatism, 35 to 40% have moderate astigmatism (>1 diopter), and 22.2% have astigmatism > 1.5 diopters [3,4].

The calculation of astigmatism correction is more complex than the spherical components because astigmatism comprises two polar components: magnitude and direction. The spherical elements of refractive errors, namely myopia and hyperopia, can be modified using arithmetic addition or subtraction. This is not the case for astigmatism since the polar values must be converted to vectors in a Cartesian plane to achieve astigmatism modification with surgery. Finally, astigmatism’s direction may be grouped by axis, with the rule or against the rule, and it can also be classified as regular or irregular, depending on the morphology of the principal topographic meridian. We did not consider internal astigmatism (the difference between refractive astigmatism and corneal astigmatism) since it has been attributed to the refracting power of the lens, and this study only includes pseudophakic patients.

All of the above helps understand the complexity of estimating the power of IOLs used for astigmatism correction, also known as toric IOLs. Additionally, another factor to take into account is the fact that the cornea has two surfaces. For years, toric IOLs were calculated exclusively considering the difference in power between the steepest and the flattest meridian of the anterior corneal surface because, based on Snell’s Law, the posterior corneal surface was considered a constant value. However, it was later observed that the astigmatic value of the posterior corneal surface is inconsistent, so it must be adjusted, either in a customized way or using nomograms, to the value of the anterior surface. In this regard, controversy remains about the accuracy of using nomograms in posterior surface adjustment compared to posterior surface individualization. The nomograms are generated from a database that includes the values analyzed of a determined number of corneas of different ages and sexes, generating reference values. On the contrary, the individualization of the posterior surface that does not consider the nomograms can adjust the corneal anterior surface in a personalized way.

The high prevalence of astigmatism in the general population and the need to obtain predictable results in the more complex toric IOL calculation prompted the creation of different formulas that consider posterior and not only anterior corneal astigmatism [5]. Among the options available, some of the most recent formulas considering total astigmatism are the Barrett, enVista, and Panacea toric calculators. The Barret toric calculator calculates recommended IOL and refractive outcomes according to the Barrett Universal II Formula. The Barrett Universal II formula evolved from the Barrett Universal I, published by Graham Barrett in 1987, and is the most widely used and trusted formula [6]. The Panacea calculator, developed by David Flickier, is the only one that allows the inclusion of the Q value of corneal asphericity and the radius of the anterior and posterior crystalline lens. Finally, the current version of the enVista toric calculator (Bausch & Lomb, Bridgewater, NJ, USA) is a proprietary calculator that incorporates the Emmetropia Verifying Optical (EVO) toric calculator, combining surgically induced astigmatism (SIA) input with the measured preoperative anterior corneal astigmatism, predicted posterior corneal astigmatism, and effective lens position, along with other variables, to generate the predicted total corneal astigmatism.

These comprehensive formulas for toric IOL calculation were developed considering several factors not fully disclosed by their creators. Therefore, it is unknown if it is possible to obtainin more precise refractive results based on the differences between the unknown variables. Due to the importance of the precise correction of astigmatism, we designed this protocol intending to evaluate residual refractive astigmatism outcomes based on IOL calculation using the Panacea and enVista toric calculators, compared to the gold-standard Barrett toric calculator.

## 2. Materials and Methods

This retrospective study was conducted in the Department of Anterior Segment Surgery of the Fundación Hospital Nuestra Señora de la Luz in Mexico City, a tertiary eye care center. This study was approved by the Ethics and Research Committee of the Fundación Hospital Nuestra Señora de la Luz (approval number 2022S12B1), and it complied with the local country regulations and the guidelines outlined in the Declaration of Helsinki. The Ethics and Research Committee also granted a waiver of informed consent for using de-identified clinical data. As this was a retrospective review, no clinical registration was required.

We reviewed the medical records of all patients who underwent phacoemulsification with the implantation of a toric IOL from May 2022 to December 2022. We selected patients with senile cataracts and regular corneal topographic astigmatism between 1.50 D and 5.0 D who had pre- and postoperative corneal topography, biometry, and refraction measurements that were reliable and consistent due to a healthy ocular surface. Different optometrists performed refraction measurements following the standard practice in our institution of corroborating that auto-refractometer values were consistent with the refraction test. Also, patients should have no history of intra- or postoperative complications. We excluded patients with ocular surface disorders or previous refractive or intraocular surgery. Also, if there was a diagnosis in the medical chart of glaucoma, ocular trauma, pseudoexfoliation, or any other factor that could affect capsular bag stability, patients were not included in the review, as zonular instability can cause IOL rotation, adversely affecting the outcomes. Four experienced surgeons performed all surgeries.

### 2.1. Preoperative Management

All patients underwent a complete anterior segment and dilated fundus examination, including uncorrected distance visual acuity and corrected distance visual acuity, manifest refraction, slit-lamp examination, and intraocular pressure (IOP) measurement using Goldmann applanation tonometry. Axial length, pachymetry, lens thickness, anterior chamber depth, and keratometry were measured with the IOL Master 700 (Software Version 1.50; Carl Zeiss Meditec, Jena, Germany). A Pentacam^®^ HR Scheimpflug camera (Software Version 1.17; Oculus Optikgeräte GmbH, Wetzler, Germany) measured anterior and posterior corneal curvature, asphericity, and Sim K values. In all patients, we used three online calculators for toric IOL calculation: Barrett toric, enVista toric, and Panacea. In all cases, we established surgeon-induced astigmatism (SIA) of 0.5 D, with the main incision (2.4 mm) at 135°. We adjusted posterior corneal astigmatism based on the nomogram of each platform. All implanted IOLs were hydrophobic acrylic, toric monofocals, free of spherical aberrations (enVista, Bausch & Lomb, Bridgewater, NJ, USA).

### 2.2. Peri- and Postoperative Management

We used limbal–corneal slit-lamp marking, with the patient seated to prevent excyclotorsion, at the 9-o-clock and 12-o-clock meridians. In the operating room, marking was performed with a Mendez ring. Four experienced surgeons performed a conventional microcoaxial phacoemulsification technique with the patient under assisted topical anesthesia. After IOL implantation, the IOL was rotated to its definitive position by aligning corneal marks with the IOL reference marks. Examinations occurred one month after surgery to perform refraction and dilated fundus examination to corroborate axis alignment. We excluded from the analysis patients with a postoperative IOL rotation greater than 5° and visible IOL tilt or decentration.

### 2.3. IOL Calculations

When conducting this retrospective chart review, we repeated the preoperative IOL calculation with the three calculators for all patients after selecting all cases. Then, we proceeded to the analysis of the comparisons.

### 2.4. Statistical Analysis

For analyses, we used RStudio and IBM SPSS version 28 statistical packages. The distribution of normality was verified with the Shapiro–Wilk test. We report means with their corresponding standard deviations for normally distributed data sets, using parametric tests for between-group comparisons. For non-normally distributed data sets, median and interquartile ranges are informed, using non-parametric tests like Kruskal–Wallis and Bonferroni for pairwise comparisons. Each group’s refractive astigmatism prediction error corresponding to the different calculators was calculated as the difference between the actual and the predicted postoperative refractive astigmatism at the corneal plane. We used refraction values for preparing double-angle plots to describe the postoperative centroid’s mean, standard deviation, and 95% confidence ellipses. Results are expressed in diopters; a *p* < 0.05 was considered statistically significant.

## 3. Results

We included 110 eyes of 90 patients, of which 5 were excluded for having a rotation > 5°, 6 were excluded due to intraoperative complications, 8 were lost to follow-up, and 11 had incomplete records. We analyzed the remaining 80 eyes that met all inclusion and no exclusion criteria. We show the demographic characteristics of the study population in Table 1.

The frequency of preoperative astigmatism according to the axis was 70 (84%) eyes showing with-the-rule (WTR) astigmatism, 9 (14%) eyes with against-the-rule (ATR) astigmatism, and 1 (2%) eye with oblique astigmatism. An analysis of variance that compared preoperative astigmatism showed no statistically significant differences between groups (Table 2).

**Figure 1:**
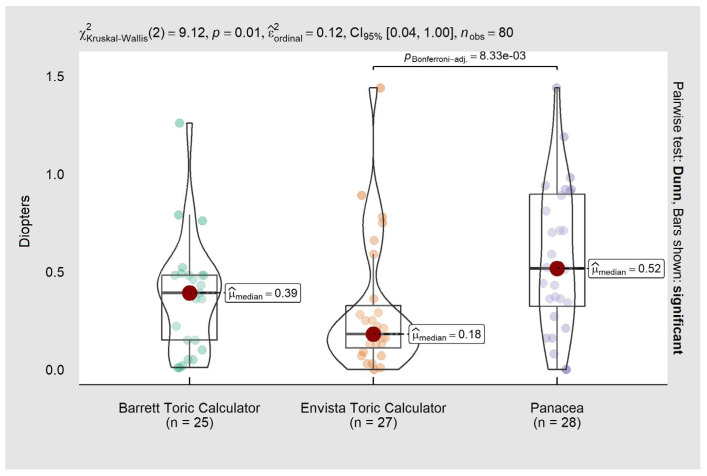
Comparison of astigmatism prediction errors.

Figure 1 shows the comparison of refractive astigmatism prediction errors. There were significant differences between calculators, with a median (interquartile range) of 0.39 (−0.13–0.48), 0.18 (0.09–0.36), and 0.52 (0.29–0.91) for Barrett, enVista, and Panacea, respectively; a pairwise comparison (*p* = 0.010) showed a statistically significant difference between enVista and Panacea.

**Figure 2:**
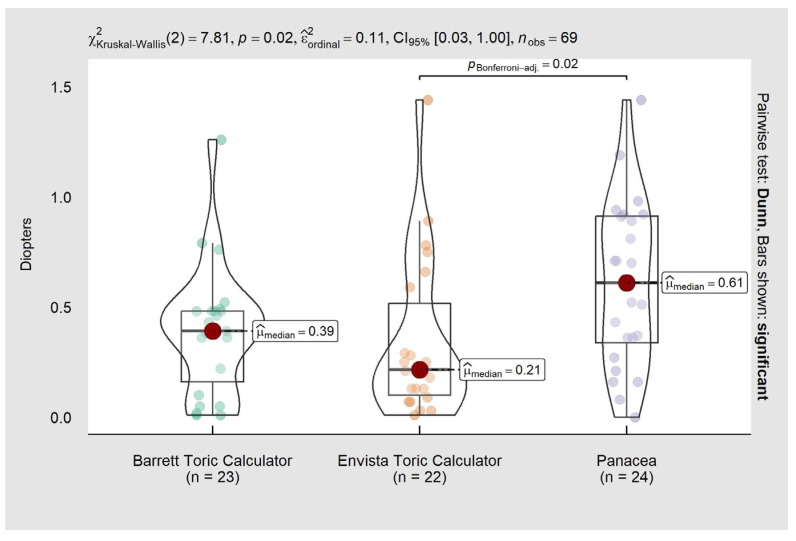
Comparison of prediction error for with-the-rule (WTR) astigmatism.

We performed a subanalysis for WTR astigmatism that indicated statistically significant differences (*p*-value 0.020). In the pairwise comparison, there were differences between the enVista (median = 0.21) and Panacea (median = 0.61) calculators (Figure 2). It was not feasible to perform a subanalysis for ATR and oblique astigmatism due to the small number of patients in each subgroup.

**Figure 3:**
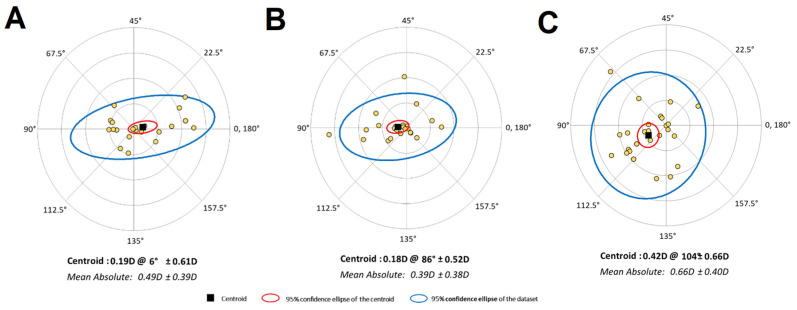
Double-angle plots of the postoperative refractive astigmatism prediction error centroid by calculator. (**A**) Barrett, (**B**) enVista, (**C**) Panacea. Abbreviations: D, diopters.

The RA prediction error centroid was similar for the Barrett and enVista toric calculators, and both were lower compared to the Panacea calculator (x-component *p* < 0.001; y-component *p* = 0.290). The RA prediction error centroid was ATR-oriented with the Barret calculator and WTR-oriented with the enVista and Panacea calculators (Figure 3).

## 4. Discussion

The last few decades have brought new technologies and knowledge about the cornea’s refractive power and its impact on the eye’s optical system, and these have been incorporated into IOL calculation. Specifically, corneal astigmatism has been extensively studied, and each discovery leads to the incorporation of new variables in available or novel formulas for toric IOL calculation. For example, calculators such as Barrett and enVista calculate toric lenses considering posterior corneal astigmatism, among other variables [7]. On the other hand, the Panacea calculator considers asphericity at 4 mm, and it uses the sum of vectors of the anterior and posterior cornea to calculate total corneal astigmatism, a method that, in theory, is more accurate compared to other calculation methods based on Gaussian optics [7].

Even though one can assume that the performance of a specific calculator may be better based on the variables it includes, it is highly relevant to objectively compare the performance of available calculators to increase the knowledge about potential outcomes and to facilitate the choice of the ideal calculator we use in our daily practice. This study compared two commonly used calculators in our institution versus the globally used Barrett calculator by measuring the differences in astigmatism refractive prediction error and the RA prediction error centroid. Our results show that the Barrett and enVista calculators have a similar performance. For Barrett, this is not surprising due to its well-known precise calculations, demonstrated and published elsewhere [8,9]; however, our positive results with the enVista calculator are inconsistent with previous studies [10] that showed less predictive precision. This improvement may be explained by the addition of the EVO Toric Formula 2.0, which has demonstrated excellent performance [11,12], to the enVista toric calculator in 2021.

Regarding centroid results, with Barrett, we observed an effect on the axis that resulted in ATR astigmatism. No studies report this outcome [13], and we cannot exclude that this axis change is due to other factors, such as changes in the vectors generated by the incision [14].

The strengths of this study are the sample size, which is larger compared to other studies that compare predictive errors [10], and the use of corneal topography and biometry measurements in all cases, allowing the capture of accurate measurements in the calculators, reducing the risk of refractive errors or surprises. We also used these measurements to perform vectorial analyses, the most valid method to evaluate residual astigmatism.

The limitations of this work are the following. First, we included the cases of four surgeons, and every surgeon has an average value of induced astigmatism. However, we aimed to standardize this figure by choosing an arbitrary value of 0.5 diopters for calculation purposes. Second, we assume some variability in postoperative refractions, as different optometrists performed them. Finally, even though a retrospective analysis of toric IOL calculations is considered a valid methodology for assessing astigmatism outcomes, we acknowledge that a prospective study would be the ideal way to evaluate the performance of different calculators.

In conclusion, this study demonstrates that, although the Barrett calculator is one of the most accurate, including the EVO in the enVista toric calculator improved the prediction of pseudophakic refractive results when implanting toric lenses.

## Figures and Tables

**Table 1:** Demographic characteristics.

Parameter	Mean ± SD
Age	64 ± 5.7 years
Sex	36 (45%) Men
	44 (55%) Women

**Table 2:** Preoperative astigmatism comparison between groups.

	Barrett	enVista	Panacea	Total
N	25	27	28	80
Mean ± SD	2.5604 ± 0.9589	2.8289 ± 1.1864	2.745 ± 1.064	2.716 ± 1.0686

Comparison between groups with a *p*-value = 0.658691 estimated with one-way ANOVA for independent measures.

## Data Availability

Data available on request from the authors.
